# Exposure to Parenting by Lying in Childhood: Associations with Negative Outcomes in Adulthood

**DOI:** 10.3389/fpsyg.2017.01240

**Published:** 2017-08-03

**Authors:** Rachel M. Santos, Sarah Zanette, Shiu M. Kwok, Gail D. Heyman, Kang Lee

**Affiliations:** ^1^Department of Applied Psychology and Human Development, University of Toronto, Toronto ON, Canada; ^2^Department of Psychology, University of California, San Diego, La Jolla CA, United States; ^3^School of Education, Zhejiang Normal University Jinhua, China

**Keywords:** parenting by lying, lying, dishonesty, psychosocial adjustment, development

## Abstract

Parents around the world engage in the practice of parenting by lying, which entails lying to manipulate children’s emotional states and behavior. The current study is the first to examine whether exposure to parenting by lying in childhood is associated with later dishonesty and psychosocial maladjustment in adulthood. Female undergraduate adults retrospectively reported their experiences of parenting by lying during childhood, the current frequency at which they lie to their parents, and their current psychosocial functioning. We found that adults who recalled relatively high levels of parenting by lying during childhood both lie to their parents more often and experience greater psychosocial adjustments problems in adulthood than adults who recalled relatively low levels of parenting by lying during childhood. This study is the first to suggest that parenting by lying during childhood may be associated with negative moral and social outcomes later in life.

## Introduction

Parents strongly believe in the importance of instilling the value of honesty in their children from a very young age (Engels et al., 2006; Bureau and Mageau, 2014). Paradoxically, parents also often lie to their children to manipulate their emotions and behaviors, a practice which is referred to in the literature as parenting by lying (Heyman et al., 2009). However, little is known as to whether parenting by lying in childhood is associated with moral or social outcomes later in life. The present study begins to fill this significant gap by examining whether exposure to parenting by lying in childhood is associated with increased dishonesty toward parents and psychosocial adjustment problems in adulthood.

Although childhood lying has been studied for many decades (see Lee, 2013 for a review), researchers have only recently become interested in parenting by lying. The first published paper on this topic was an anthropological study conducted on Tzeltal-speaking Mayan farmers in Tenejapa, a rural community located in Southern Mexico (Brown, 2002). In this study, parents frequently lied to their children to try to control their behavior. For example, farmers would lie to their children about rabid animals in the wild to discourage them from wandering off the farm.

Building on this initial work, researchers have examined the lies parents tell their children in the United States and China (Heyman et al., 2009, 2013). These studies have shown that the vast majority of parents engage in this practice (84% of parents in the United States and 98% of parents in China) and that the topics of their lies vary widely: often including lies related to eating, money, misbehavior, and false threats of abandoning the child (such as in a public place) (Heyman et al., 2013). Despite the high prevalence of parenting by lying, most parents report teaching their children that lying is unacceptable (Heyman et al., 2009). Surprisingly, the parents who reported the most negative judgments about their children lying were also the parents most likely to report lying to their own children (Heyman et al., 2009).

There is intriguing evidence suggesting there may at least be negative short-term effects when children are exposed to adults who lie: Hays and Carver (2014) and Yi et al. (2014) found that children are more likely to lie to an unfamiliar adult who first lies to them. Moreover, Yi et al. (2014) discovered that this increased tendency to lie extends to other individuals and contexts. This research suggests that when adults lie to children they may be implicitly teaching their children to lie. Even when children do not consciously recognize parents’ statements as lies when they hear them, it is possible that they might recognize them later, or that they might begin to develop a general sense that their parents cannot be relied upon. Thus, it is possible that through modeling children learn to lie to parents who lie to them and that parenting by lying in particular may be associated with more pervasive and long-term outcomes later in life.

Prior research has indicated that children’s lying is associated with a host of negative outcomes, such as an increased risk of developing externalizing and antisocial personality problems (Stouthamer-Loeber and Loeber, 1986; Gervais et al., 2000; Engels et al., 2006). For example, persistent lying in childhood is associated with disruptive behavior problems (Gervais et al., 2000) and problem behaviors such as theft, delinquency, and fighting (Stouthamer-Loeber and Loeber, 1986). Similarly, adolescents who frequently lie to their parents often have problems with aggression, delinquency, and self-control (Engels et al., 2006). Less is known about the potential relationship between lying and internalizing problems, but there is some evidence to suggest that more frequent lying to parents is related to increased depressive mood, feelings of alienation, and reduced trust toward parents and peers (Engels et al., 2006). Overall, existing research demonstrates a relation between lying and externalizing and internalizing behavior problems, highlighting the importance of research regarding factors that may contribute to increased lie-telling.

The goal of the current study was to take a first step in investigating the moral and social associations of exposure to parenting by lying during childhood. To do so, female undergraduate adults completed three self-report questionnaires measuring their recollection of experiencing parenting by lying in their childhood, the current frequency at which they lie to their parents, and their current psychosocial functioning (i.e., internalizing, externalizing, and antisocial personality problems). Based on the existing, albeit limited, evidence, we hypothesized that: (1) greater recall of experiencing parenting by lying in childhood will be related to more frequent lying to parents in adulthood; (2) more frequent lying to parents in adulthood will be associated with greater psychosocial maladjustment; and (3) recalling greater exposure to parenting by lying in childhood will predict greater psychosocial maladjustment in adulthood.

## Materials and Methods

### Participants

Fifty female undergraduate students participated in the current study (*M*_age_ = 20.73 years, *SD* = 4.83 years). We limited our sample to females due to difficulty in recruiting male participants. Participants were recruited from a large North American university through online advertisements and flyers posted throughout campus. The ethnic composition of our sample reflected the diversity of the city from which the participants were recruited. According to self-report data, 63% of the participants were Asian, 16% were White, 3% were Black, 2% were bi-racial/multiethnic, 2% were Hispanic, 11% indicated another ethnicity, and two participants (3%) chose not to indicate their ethnicity. Participants were compensated $5.00 for their time.

### Procedure and Materials

Prior to participation, participants were provided with a letter of information and a consent form detailing the nature of the research objectives, procedure, and potential risks and benefits of participation. Once informed consent was obtained, participants independently completed three questionnaires (described below) in a quiet room on campus. The nature, procedure, and methods used in this study were approved by the university’s research ethics board and all regulatory standards were upheld during data collection.

#### Parenting by Lying Questionnaire

The parenting by lying questionnaire, adapted from Heyman et al. (2013), is a 16-item questionnaire assessing how many types of lies adults remember their parents saying to them during childhood. Since this is the first study to examine the potential relationship between parenting by lying in childhood and negative outcomes in adulthood, we chose not to restrict participant’s recall of parenting by lying to a specific period within childhood, and instead focus on experiences in childhood more broadly.

The parenting by lying questionnaire is based on four categories of lies commonly told to children by parents: (1) lies related to eating, *“you need to finish all your food or you will get pimples all over your face”*; (2) lies related to leaving/staying, *“if you do not come with me now, I will leave you here by yourself”*; (3) lies related to misbehavior, *“if you do not behave, I will call the police”*; and (4) lies related to money, *“we do not have enough money to buy that toy.”* Participants were asked to read each lie (four items/lies per lie-category) and indicate whether they remember their parent telling that lie to them as a child by indicating *“yes,” “no,”* or *“I don’t know.*”

Following the coding scheme used in Heyman et al. (2013), we generated one score for each of the four lie categories. A score of 1 was awarded if the participant indicated “yes” to at least one of the four lies within a category: otherwise, they were given a score of 0. We then summed the four category scores together to create a total parenting by lying score. Scores ranged from 0 (participant did not recall being told any of the lies indicated) to 4 (participant remembers being told at least one lie within each of the four categories), with higher parenting by lying scores indicating relatively greater exposure to this practice. Reliability for this measure is considered acceptable with good internal consistency (α = 0.70).

#### Lying to Parents Questionnaire

The lying to parents questionnaire, adapted from Engels et al. (2006), is a 12-item measure used to determine the frequency of lying to parents. Specifically, the lying to parents questionnaire measures the frequency of three types of lies told to parents: antisocial lies (e.g., lying to conceal a transgression; eight items), prosocial lies (i.e., lying to prevent hurting someone’s feelings; two items), and lying by exaggeration (two items). Sample items include: how often do you *“…lie to your parents about what you do with your friend*s” (antisocial lying), *“…sometimes do not tell the truth so you do not have to hurt someone else’s feeling*s” (prosocial lying), and *“…exaggerate to your parents about the things you experience”* (lying by exaggeration). Participants rated each statement on a 5-point Likert scale ranging from 0 = *never* to 4 = *very often.* The 12 items were summed together to create a total participant lie score, referred to henceforth as “lying to parents,” with higher scores indicating more frequent lying to parents. A total lying score was used to better understand the global nature of participant lying to parents. Reliability for this measure is acceptable with high internal consistency (α = 0.90).

#### Adult Self-report (ASR) Questionnaire

The ASR is a 126-item questionnaire measuring adults’ overall adaptive functioning (e.g., relationships with others) and behavioral, emotional, and social problems based on the DSM-V scales (Diagnostic and Statistical Manual of Mental Disorders) (Achenbach, 2003). For the purpose of this study, we used scores (gender normed for ages 18–59) generated by the ASR to measure three indicators of psychosocial maladjustment: internalizing problems (e.g., anxiety, depression, withdrawn behavior; α = 0.93), externalizing problems (e.g., aggression, rule-breaking, intrusive behavior; α = 0.89), and antisocial personality problems (e.g., damage to property, lack of guilt, cheating; α = 0.74). Higher scores on these scales indicate more severe problems in that area.

## Results

The goal of the current study was to determine if remembering experiences of parenting by lying in childhood is associated with lying to parents and psychosocial maladjustment in adulthood. To evaluate this possibility, we conducted a series of linear regressions to explore relationships between: (1) exposure to parenting by lying in childhood and the frequency of lying to parents in adulthood; (2) the frequency of lying to parents in adulthood and psychosocial maladjustment (internalizing, externalizing, and antisocial personality problems); and (3) exposure to parenting by lying in childhood and psychosocial maladjustment in adulthood. Finally, we conducted mediation analyses, with the frequency of lying to parents in adulthood as a mediator, to try to explain the significant associations uncovered between exposure to parenting by lying in childhood and psychosocial maladjustment in adulthood.

### Parenting by Lying and the Frequency of Lying to Parents

We conducted a simple linear regression to determine whether parenting by lying is associated with the frequency of lying to parents. Parenting by lying was entered as the predictor variable and lying to parents served as the dependent variable. Parenting by lying significantly predicted lying to parents, explaining 8% of the total variance, Δ*R*^2^ = 0.08, Δ*F*(1,48) = 4.33, *p* = 0.043. Thus, as exposure to parenting by lying in childhood increased, the frequency of lying to parents during adulthood also increased, *b*_parent lying_ = 0.29, *SE* = 0.89, *t*(49) = 2.08, *p* = 0.043, 95% CI [0.06, 3.65], *r*_part_ = 0.29.

### Frequency of Lying to Parents and Psychosocial Maladjustment

To determine whether the frequency of lying to parents in adulthood is associated with psychosocial maladjustment, we conducted three separate simple linear regressions. For each regression, the frequency of lying to parents in adulthood was entered as the predictor variable and one of the three psychosocial maladjustment variables (internalizing, externalizing, or antisocial personality problems) was entered as the dependent variable.

Results for all three regressions demonstrated a significant positive linear relationship with the frequency of lying to parents in adulthood (see **Table 1**; step 1). That is, as the frequency of lying to parents increased, the severity of psychosocial adjustment problems also increased. Specifically, lying to parents explained 26% of the variance in internalizing problems, 31% of the variance in externalizing problems, and 36% of the variance in antisocial personality problems.

**Table 1:** Lying to parents and psychosocial adjustment problems.

Adjustment problem	IVs	B	SE B	β	*t*	*p*	95% CI	*r*_part_	*R*^2^	Δ*R*^2^	Δ*F*^2^	Sig. Δ*F*^2^
Internalizing	Step 1											
	Lying to parents	0.73	0.19	0.51	3.96	<0.001	[0.36, 1.10]	0.51	0.26	–	15.72	<0.001
	Step 2											
	Parental lying	1.38	1.22	0.15	1.13	0.267	[-1.09, 3.84]	0.14	0.28	0.02	1.27	0.267
Externalizing	Step 1											
	Lying to parents	0.74	0.17	0.56	4.48	<0.001	[0.41, 1.07]	0.56	0.31	–	20.04	<0.001
	Step 2											
	Parental lying	1.95	1.07	0.23	1.82	0.075	[-0.21, 4.10]	0.22	0.36	0.05	3.32	0.075
Antisocial personality	Step 1											
	Lying to parents	0.47	0.09	0.60	5.06	<0.001	[0.28, 0.66]	0.60	0.36	–	25.65	<0.001
	Step 2											
	Parental lying	1.37	0.59	0.28	2.33	0.025	[0.18, 2.56]	0.26	0.43	0.07	5.42	0.025


### Parenting by Lying and Psychosocial Maladjustment

Next, we examined whether exposure to parenting by lying in childhood is related to psychosocial maladjustment in adulthood. Given the significant relations found between the frequency of lying to parents and psychosocial adjustment problems, we conducted three hierarchical linear regressions with the frequency of lying to parents in adulthood entered on the first step and parenting by lying in childhood entered on the second step. In each regression, one of the three psychosocial adjustment variables acted as the dependent variable.

As shown in **Table 1** (step 2), after controlling for the frequency of lying to parents in adulthood, exposure to parenting by lying in childhood did not uniquely account for a significant portion of the variance in internalizing problems or externalizing problems. However, exposure to parenting by lying in childhood significantly and uniquely accounted for 7% of the variance in antisocial personality problems in adulthood. These results indicate that as parenting by lying scores increase, antisocial personality problems in adulthood also increase, even after controlling for the frequency of lying to parents.

### Explaining the Relationship between Parenting by Lying and Maladjustment

**FIGURE 1:**
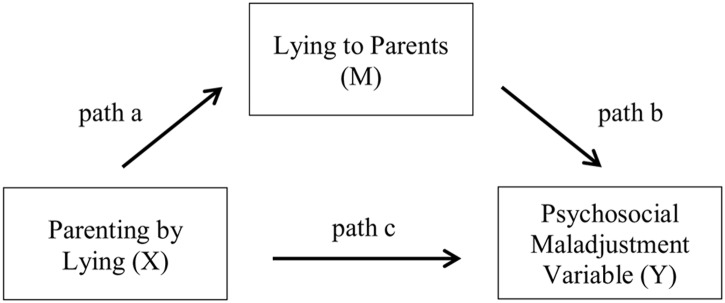
Conceptual mediation model for psychosocial adjustment problems.

Using the Preacher and Hayes (2004, 2008) statistical method of mediation analysis, along with recommendations from Zhao et al. (2010), we examined the direct and indirect effects among exposure to parenting by lying during childhood, the frequency of lying to parents in adulthood, and self-reported psychosocial maladjustment in adulthood. Specifically, we examined whether the frequency of lying to parents in adulthood (mediator: M) influences the relationship between parenting by lying (independent variable: *X*) and self-reported internalizing, externalizing, and antisocial personality problems (dependent variables: *Y*) (see **Figure 1**).

We explored the presence of a mediation by first determining whether an indirect relation exists between the independent and dependent variable of interest through the mediator (indirect effect: *path a × b*). We then examined whether the independent variable also influences the dependant variable directly (direct effect: *path c*, after controlling for the potential mediator) (Preacher and Hayes, 2004, 2008; Zhao et al., 2010). Based on these results, we can then classify the type of mediation as follows: (1) *direct-only mediation*: the indirect path is not significant but the direct path is; (2) *indirect-only mediation*: the indirect path is significant but the direct path is not; (3) *complementary mediation*: both the indirect and direct paths are significant and occur in the same direction (both positive or both negative coefficients); and (4) *competitive mediation*: both the indirect and direct paths are significant but occur in opposite directions (one positive and one negative coefficient) (Zhao et al., 2010).

Unlike in the Baron and Kenny (1986) approach to mediation, Zhao et al. (2010) argue that a significant path c is not required for a mediation to exist (see Zhao et al., 2010 for details). Thus, we were able to conduct mediation analyses for all three psychosocial maladjustment variables.

#### Parenting by Lying and Internalizing Problems

As discussed above, we found significant relationships between exposure to parenting by lying and lying to parents in adulthood (path a), and lying to parents in adulthood and internalizing problems (path b; **Table 1**). Parenting by lying and internalizing problems were not significantly related [path c; *b* = 2.60, *t* = 1.99, Δ*R*^2^ = 0.08, Δ*F*(1,45) = 3.96, *p* = 0.053, part correlation = 0.28].

**FIGURE 2:**
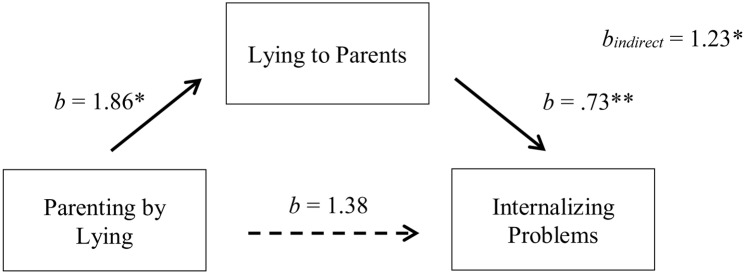
Indirect-only mediation model between parenting by lying and internalizing problems. The dotted line represents the bootstrapped direct effect of X on Y after controlling for M. ^∗^*p* < 0.05, ^∗∗^*p* < 0.001.

After bootstrapping 5000 times (Preacher and Hayes, 2004, 2008), we found a significant indirect effect between parenting by lying and internalizing problems through lying to parents (path a × b; *b* = 1.23, *SE* = 0.56, 95% CI [0.35, 2.56]), explaining 53% of the total effect. After controlling for lying to parents, we found no significant direct effect of parenting by lying on internalizing problems (*b* = 1.38, *SE* = 1.22, 95% CI [-1.09, 3.84]) (**Figure 2**). Together, these results indicate that an indirect-only mediation occurs between parenting by lying in childhood and internalizing problems in adulthood, with lying to parents acting as a mediator (Zhao et al., 2010).

#### Parenting by Lying and Externalizing Problems

**FIGURE 3:**
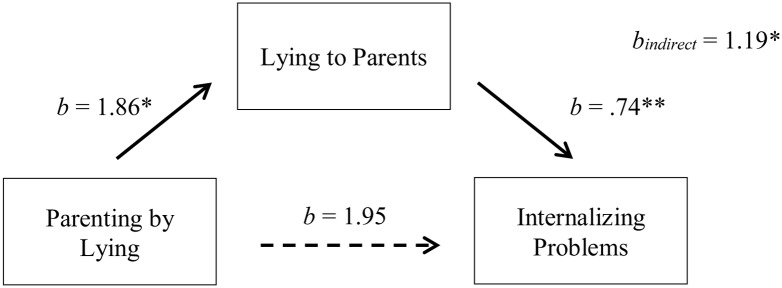
Indirect-only mediation model between parenting by lying and externalizing problems. The dotted line represents the bootstrapped direct effect of X on Y after controlling for M. ^∗^*p* < 0.05, ^∗∗^*p* < 0.001.

As mentioned above, we found significant relations between exposure to parenting by lying and lying to parents in adulthood (path a), lying to parents in adulthood and externalizing problems (path b; **Table 1**), and parenting by lying and externalizing problems [path c; *b* = 3.14, *t* = 2.68, Δ*R*^2^ = 0.14, Δ*F*(1,45) = 7.17, *p* = 0.010, part correlation = 0.37] (see **Figure 3**).

After bootstrapping 5000 times (Preacher and Hayes, 2004, 2008), we found a significant indirect effect between parenting by lying and externalizing problems through lying to parents (path a × b; *b* = 1.19, *SE* = 0.52, 95% CI [0.31, 2.39]), explaining 44% of the total effect. After controlling for lying to parents, we found no significant direct effect of parenting by lying on externalizing problems (*b* = 1.95, *SE* = 1.07, 95% CI [-0.21, 4.10]) (**Figure 3**). Thus, similar to internalizing problems, these results indicate that an indirect-only mediation occurs between parenting by lying in childhood and externalizing problems in adulthood, with lying to parents acting as a mediator (Zhao et al., 2010).

#### Parenting by Lying and Antisocial Personality Problems

In addition to the significant relations between exposure to parenting by lying and lying to parents in adulthood (path a) and lying to parents in adulthood and antisocial personality problems (path b; **Table 1**), we uncovered a significant relationship between parenting by lying and antisocial personality problems [path c; *b* = 2.12, *t* = 3.16, Δ*R*^2^ = 0.18 Δ*F*(1,45) = 10.00, *p* = 0.003, part correlation = 0.43].

**FIGURE 4:**
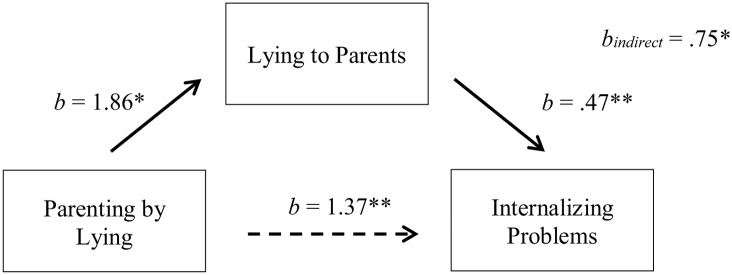
Indirect-only mediation model between parenting by lying and externalizing problems. The dotted line represents the bootstrapped direct effect of X on Y after controlling for M. ^∗^*p* < 0.05, ^∗∗^*p* < 0.001.

After bootstrapping 5000 times (Preacher and Hayes, 2004, 2008), we found a significant indirect effect between parenting by lying and antisocial personality problems through lying to parents (path a × b*; b* = 0.75, *SE* = 0.35, 95% CI [0.20, 1.61]), explaining 41% of the total effect. We also found a significant direct effect between parenting by lying and antisocial personality problems (*b* = 1.37, *SE* = 0.59, 95% CI [0.18, 2.56]) (**Figure 4**). Both the direct and indirect pathways contained positive coefficients, thus indicating that a complementary mediation exists between parenting by lying in childhood and antisocial personality problems in adulthood, with lying to parents acting as a mediator (Zhao et al., 2010).

## Discussion

Although parenting by lying is a common practice (Heyman et al., 2009, 2013), almost nothing is known about its associated outcomes. The goal of the current study was to begin to fill this gap by examining the relations between exposure to parenting by lying during childhood and moral and social outcomes later in life. We obtained four major findings. First, we found that remembering greater exposure to parenting by lying in childhood is associated with increased dishonesty toward parents in adulthood. Second, this heightened dishonesty toward parents is associated with greater internalizing, externalizing, and antisocial personality problems. Third, recalling greater parenting by lying has unique associations with antisocial personality problems in adulthood above and beyond the contribution of lying to parents. Fourth, lying to parents in adulthood plays a mediating role between parenting by lying in childhood and psychosocial adjustment problems. We discuss these major findings in turn below.

Our first major finding was that adults who report remembering greater exposure to parenting by lying in childhood also report lying to their parents more frequently in adulthood. This finding supports prior work suggesting that observational learning may be one developmental mechanism in which children learn to lie (Hays and Carver, 2014; Yi et al., 2014). In these studies, children were more likely to lie if they had previously observed an unfamiliar adult lie to them. This could be because children learn behaviors through direct observation of their parents (Bandura, 1977). Previous work on this topic in the sociomoral domain has largely been limited to children’s willingness to engage in aggressive (Bandura, 1965) and prosocial behavior (Elliott and Vasta, 1970). Our findings suggest that observational learning may also be important in the socialization of lying. Specifically, we found that young adults who are able to retrospectively recognize and remember their parents having lied to them are more likely to lie to their parents in adulthood. This suggests that parents who lie to their children as a means of manipulating their children’s emotions and behavior may be inadvertently modeling that lying is an acceptable way to communicate and solve problems (see Hays and Carver, 2014; Yi et al., 2014 for similar arguments).

Our second major finding was that young adults’ lying to parents was positively correlated with their internalizing, externalizing, and antisocial personality problems. This finding is consistent with prior research involving children, adolescents, and young adults, demonstrating that frequent lying in childhood is associated with negative psychosocial outcomes (Engels et al., 2006; Perkins and Turiel, 2007; Ennis et al., 2008). These associations are particularly strong when parents are the targets of the dishonesty (Jensen et al., 2004; Smetana et al., 2009; Cumsille et al., 2010); frequent lying to parents is associated with a host of internalizing (e.g., depression and/or emotional difficulties), externalizing (e.g., aggression), and antisocial personality problems in adolescence. The results of the current study further contribute to evidence suggesting that frequent dishonesty may be a negative indicator of social and emotional well-being by providing further evidence that frequent lying to parents is associated with the aforementioned psychosocial problems in adulthood. However, further research will be needed to understand the nature of this relationship and to explore alternative explanations. For example, psychosocial adjustment in adulthood could affect memories of parental lying and stable personality traits could affect memories of parental lying and psychosocial problems.

One explanation for the observed relation between increased dishonesty and psychosocial adjustment problems in the present study pertains to the negative emotions dishonesty can elicit (Jensen et al., 2004). For example, lying to one’s parents may trigger feelings of guilt and regret (internalizing problems), anger and aggression (externalizing problems), and the development of antisocial personality problems, such as violating the rights of others. However, it is also possible that psychosocial adjustment problems contribute to greater dishonesty. For example, a child who frequently breaks the rules (externalizing problem) may resort to more frequent lie-telling in order to hide this misbehavior. Future research should explore this topic to uncover the nature of this relationship.

A third major finding was that parenting by lying had unique associations with antisocial personality problems in adulthood, but not with internalizing or externalizing problems. This finding was based on the regression analyses of psychosocial adjustment problems in adulthood after controlling for the frequency of lying to parents. The unique relation between remembering greater exposure to parenting by lying and the development of antisocial personality problems may be because parents who choose to lie to their children could be missing key opportunities for teaching their child appropriate problem solving and conflict resolution skills. Without obtaining these skills, children are at risk for developing a range of problem behaviors such as socially deviant attitudes and behaviors (Colsman and Wulfert, 2002).

Our fourth major finding was the uncovering of indirect-only and complementary mediations between parenting by lying and psychosocial maladjustment. According to our mediation analyses of the relationships between parenting by lying in childhood and psychosocial maladjustment, we found that indirect-only mediations exist between parenting by lying and both internalizing and externalizing problems, with lying to parents serving as the mediator. This indirect pathway suggests that greater parenting by lying is associated with more frequent lying to parents in adulthood, which in turn is related to both internalizing and externalizing problems. Thus, it may be that participants who remember observing parenting by lying during childhood learn from parents that lying is a permissible method of achieving a desirable goal and, as a result, practice this behavior by lying to their parents in adulthood. Lying to one’s parents may then evoke both internal feelings of guilt and regret (internalizing problems) and external actions of anger and hostility (externalizing problems), thereby demonstrating an indirect relationship between parenting by lying and these specific maladjustments.

Additionally, we found that both direct and indirect pathways occur between parenting by lying and antisocial personality problems. As discussed above, the direct pathway may reflect a socialization environment where there is a lack of opportunity for learning conflict resolution skills, thus inadvertently promoting the development of antisocial personality problems (e.g., aggression). However, we found that the associations between parenting by lying and participants’ antisocial personality problems also exist through an indirect pathway between participants’ lying to their parents. Similar to the interpretation above, this suggests that parenting by lying may affect the development of antisocial personality problems through participants’ tendency to lie to their parents in adulthood. It may be that children who are exposed to more parenting by lying not only implicitly learn that it is okay to lie to family members, but also generalize this understanding to other behaviors and situations, such as aggression, disobeying rules, and cheating. This may also help explain why lying to parents in adulthood mediates the relationship between parenting by lying in childhood and externalizing problems in adulthood.

The present study has several limitations that should be addressed in future research. One major limitation is the relatively small sample size that primarily consisted of Asian female adults. Future research will be needed to examine the present research questions in a larger, more representative gender-balanced sample. Including male adults will be important given that males are more likely to experience externalizing problems, whereas females are more likely to experience internalizing problems (Zahn-Waxler et al., 2008). Additionally, research suggests that male adolescents are more tolerant of lying (Keltikangas-Järvinen and Lindeman, 1997) and lie to parents more often than females (Engels et al., 2006).

An additional significant limitation is that we relied on the retrospective reports of young adults to assess parenting by lying. Doing so means that there are likely memory errors that add noise to our data. Although we attempted to mitigate such errors by instructing our adult participants to only answer the items in which they were confident about, the potential for memory errors in this study could not be eliminated. An additional problem with using retrospective reports is that individuals cannot accurately report on parenting by lying without recognizing the lies their parents told at the time or after the fact. It is likely that parents who lie frequently have children who recognize statements as lies more frequently, but this is an empirical question. Having researchers observe parent–child interactions has the potential to resolve some of these issues, such as examining lies that children might not be aware of. However, this method would also likely to lead to difficulties assessing which statements are lies given that parents sometimes make false statements for reasons that are not lies (e.g., based on their false beliefs) and that researchers may have difficulty assessing whether some statements are true (e.g., descriptions of previous parent–child interactions).

Future research should also involve child participants to directly assess how parenting by lying affects psychosocial adjustment during childhood. It would be best if such a study incorporated a longitudinal design to investigate parenting by lying throughout development. Future research is also needed to explore other possible associations between parenting by lying and psychosocial development, including associations with cognitive variables, such as how children evaluate evidence (Woolley et al., 2004; Harris et al., 2006; Woolley and Ghossainy, 2013). Additionally, it will be important to determine whether different forms of lying, such as lying to protect the child’s feelings (i.e., prosocial lying), have different associations with behavior and adjustment. Finally, future research will be needed to examine how the present findings generalize across different cultures.

## Conclusion

The present study is the first to investigate associations between exposure to parenting by lying in childhood and psychosocial development into adulthood. We discovered that remembering greater exposure to parenting by lying in childhood is associated with increased dishonesty toward parents in adulthood and with the development of internalizing, externalizing, and antisocial personality problems. These findings point to the need for further research on understanding the mechanisms that can account for these effects.

## Ethics Statement

The following study was approved by the University of Toronto Research and Innovation department. As per ethical protocol, all research participants were guided through an informed consent process in which they were made aware of the purpose of the study, potential risks and benefits to participating, and the right to withdrawal consent. Participants provided written consent to participate. Additionally, all participants were compensated for their time. No exclusions were made and vulnerable populations were not used.

## Author Contributions

The execution of this paper was truly a combined effort on behalf of all authors. KL developed the original study idea and concept. RS expanded the study concept, selected the appropriate measures, and designed the study. SK collected and entered the majority of the data used for this study. KL, RS, and SZ conducted the subsequent data analyses and interpretations. RS drafted the initial manuscript before SZ, GH, and KL contributed to the writing of the manuscript. All authors approved the final version of the paper for submission.

## Conflict of Interest Statement

The authors declare that the research was conducted in the absence of any commercial or financial relationships that could be construed as a potential conflict of interest.
