# Corrigendum: Exposure to Parenting by Lying in Childhood: Associations with Negative Outcomes in Adulthood

**DOI:** 10.3389/fpsyg.2017.01900

**Published:** 2017-10-26

**Authors:** Rachel M. Santos, Sarah Zanette, Shiu M. Kwok, Gail D. Heyman, Kang Lee

**Affiliations:** ^1^Department of Applied Psychology and Human Development, University of Toronto, Toronto, ON, Canada; ^2^Department of Psychology, University of California, San Diego, La Jolla, CA, United States; ^3^School of Education, Zhejiang Normal University, Jinhua, China

**Keywords:** parenting by lying, lying, dishonesty, psychosocial adjustment, development

In the original article, there were mistakes in Figures 2–4 as published. The psychosocial maladjustment label in Figures 3, 4 were incorrect. The corrected Figures appear below. Additionally, the authors have included the standardized beta coefficients in all three of the mediation figures.

**Figure 2:**
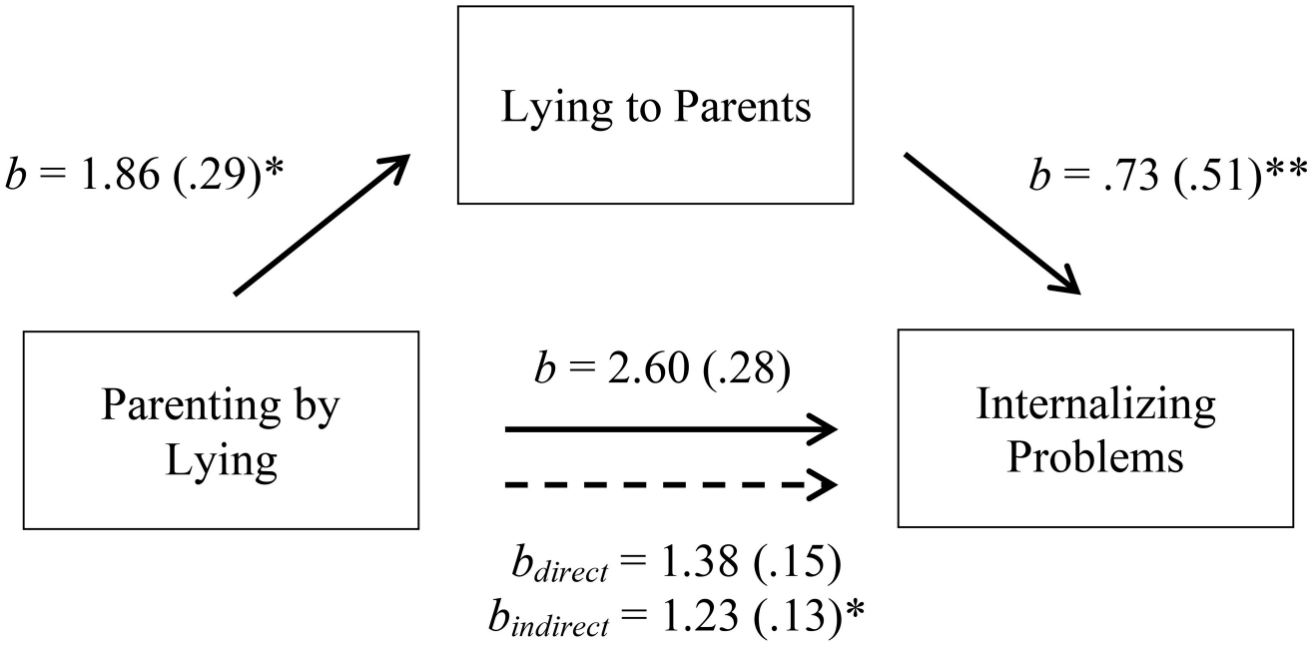
Indirect-only mediation model between parenting by lying and internalizing problems. The solid lines represent the simple linear regressions (paths a, b, and c); the dotted line represents the bootstrapped direct and indirect effects of X on Y after controlling for M. Bracketed values indicate the standardized Beta coefficient. *p < 0.05, **p < 0.001.

**Figure 3:**
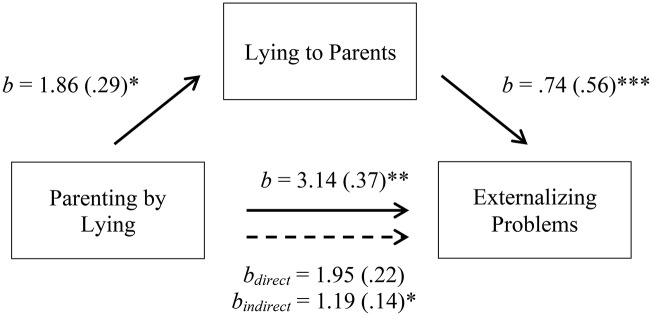
Indirect-only mediation model between parenting by lying and externalizing problems. The solid lines represent the simple linear regressions (paths a, b, and c); the dotted line represents the bootstrapped direct and indirect effects of X on Y after controlling for M. Bracketed values indicate the standardized Beta coefficient. *p < 0.05, **p < 0.01, ***p < 0.001.

**Figure 4:**
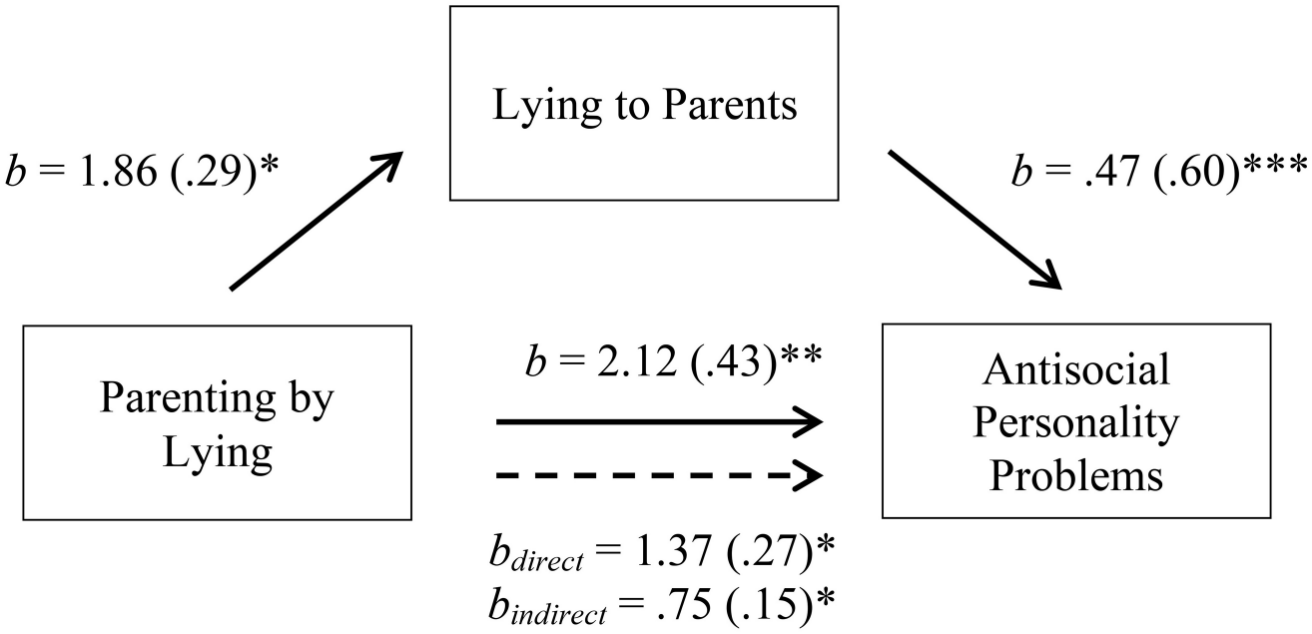
Complementary mediation model between parenting by lying and antisocial personality problems. The solid lines represent the simple linear regressions (paths a, b, and c); the dotted line represents the bootstrapped direct and indirect effects of X on Y after controlling for M. Bracketed values indicate the standardized Beta coefficient. *p < 0.05, **p < 0.01, ***p < 0.001.

Figure 2 Indirect-only mediation model between parenting by lying and internalizing problems. The solid lines represent the simple linear regressions (paths a, b, and c); the dotted line represents the bootstrapped direct and indirect effects of X on Y after controlling for M. Bracketed values indicate the standardized Beta coefficient. ^*^p < 0.05, ^**^p < 0.001.

Figure 3 Indirect-only mediation model between parenting by lying and externalizing problems. The solid lines represent the simple linear regressions (paths a, b, and c); the dotted line represents the bootstrapped direct and indirect effects of X on Y after controlling for M. Bracketed values indicate the standardized Beta coefficient. ^*^p < 0.05, ^**^p < 0.01, ^***^p < 0.001.

Figure 4 Complementary mediation model between parenting by lying and antisocial personality problems. The solid lines represent the simple linear regressions (paths a, b, and c); the dotted line represents the bootstrapped direct and indirect effects of X on Y after controlling for M. Bracketed values indicate the standardized Beta coefficient. ^*^p < 0.05, ^**^p < 0.01, ^***^p < 0.001.

In the original article, the standardized Beta value was reported in the text where the unstandardized b value should have been reported.

A correction has been made to the Results section, *Parenting by Lying and the Frequency of Lying to Parents*, paragraph one:

## Parenting by lying and the frequency of lying to parents

We conducted a simple linear regression to determine whether parenting by lying is associated with the frequency of lying to parents. Parenting by lying was entered as the predictor variable and lying to parents served as the dependent variable. Parenting by lying significantly predicted lying to parents, explaining 8% of the total variance, Δ*R*^2^ = 0.08, Δ*F*_(1, 48)_ = 4.33, *p* = 0.043. Thus, as exposure to parenting by lying in childhood increased, the frequency of lying to parents during adulthood also increased, *b*_parent lying_ = 1.86, *SE* = 0.89, *t*_(49)_ = 2.08, *p* = 0.043, 95% CI [0.06, 3.65], *r*_part_ = 0.29.

The authors apologize for these errors and state that this does not change the scientific conclusions of the article in any way.

### Conflict of interest statement

The authors declare that the research was conducted in the absence of any commercial or financial relationships that could be construed as a potential conflict of interest.

